# A barrier against reactive oxygen species: chitosan/acellular dermal matrix scaffold enhances stem cell retention and improves cutaneous wound healing

**DOI:** 10.1186/s13287-020-01901-6

**Published:** 2020-09-07

**Authors:** Wei Lin, Xiaoyang Qi, Wenjing Guo, Danyang Liang, Heting Chen, Baoping Lin, Xiaoyuan Deng

**Affiliations:** 1 MOE Key Laboratory of Laser Life Science, College of Biophotonics & Institute of Laser Life Science, South China Normal University, Guangzhou, 510631 China; 2 Guangdong Provincial Key Laboratory of Laser Life Science, College of Biophotonics, South China Normal University, Guangzhou, 510631 China; 3 The Brain Cognition and Brain Disease Institute of Shenzhen Institutes of Advanced Technology, Chinese Academy of Sciences, Shenzhen, 518055 China; 4 Guangzhou Institutes of Biomedicine and Health, Chinese Academy of Sciences, Guangzhou, 510530 China

**Keywords:** Chitosan/Acellular dermal matrix scaffold, ROS barrier in inflammatory response, Stem cell retention, Wound healing

## Abstract

**Background:**

Stem cell therapies have gained great attention for providing novel solutions for treatment of various injuries and diseases due to stem cells’ self-renewal, ability to differentiate into various cell types, and favorite paracrine function. Nevertheless, the low retention of transplanted stem cell still limits their clinical applications such as in wound healing in view of an induced harsh microenvironment rich in reactive oxygen species (ROS) during inflammatory reactions.

**Methods:**

Herein, a novel chitosan/acellular dermal matrix (CHS/ADM) stem cell delivery system is developed, which is of great ROS scavenging activity and significantly attenuates inflammatory response.

**Result:**

Under ROS microenvironment, this stem cell delivery system acts as a barrier, effectively scavenging an amount of ROS and protecting mesenchymal stem cells (MSCs) from the oxidative stress. It notably regulates intracellular ROS level in MSCs and reduces ROS-induced cellular death. Most importantly, such MSCs delivery system significantly enhances in vivo transplanted stem cell retention, promotes the vessel growth, and accelerates wound healing.

**Conclusions:**

This novel delivery system, which overcomes the limitations of conventional plain collagen-based delivery system in lacking of ROS-environmental responsive mechanisms, demonstrates a great potential use in stem cell therapies in wound healing.

## Background

There is a great potential for stem cell-based therapies to treat a myriad of diseases and injuries by means of stem cells’ ability to modulate immune or inflammatory responses through releasing trophic factors [1–3]. However, the low engraftment rates and low retention of transplanted stem cells, caused by lacking of an appropriate 3D supporting matrix for efficient stem cells delivery as well as survival microenvironment providing for stem cells, are still the critical factors that limit their clinical applications in wound healing [4]. To address this issue, scaffolds have been proposed as an effective strategy to deliver stem cells to the desired site of repair, which offer stem cells a structural support and a bio-functionally living microenvironment for cell adhesion, migration and proliferation [5]. In recent years, two major scaffold materials have been investigated, the artificial and natural materials. Natural materials usually have better biocompatibility and biodegradability compared to artificial materials. Natural collagen, which has excellent biocompatibility and enzymatic degradability, becomes one of the most popular biomaterials being widely explored as implantable scaffold [6, 7]. Acellular dermal matrix (ADM), derived from dermal extracellular matrix (ECM) of human or animal skin by decellularization process, is so far the popular applicable collagen-based bioscaffold. It is mainly composed of collagen proteins, some other remaining functional proteins such as fibronectin, laminin, and small amount of glycoproteins, glycosaminoglycans, proteoglycans, etc. ADM has a completely analogous structure and components to natural dermal skin; therefore, it has remarkable biocompatibility and biodegradability. Also, ADM is capable of providing a 3D matrix with favorable bio-structural and bio-functional properties like mechanical strength for mesenchymal stem cells (MSCs) survival. So ADM has been used as a powerful vehicle for stem cell delivery to enhance skin regeneration and tissue defect [8, 9].

However, due to the degradation of collagen, the collagen-based stem cell delivery system has shortcomings in coping with the inflammatory response in wound healing. Wound healing is a highly dynamic process which involves complex interactions of subtypes of infiltrating leukocyte and various resident cells [10, 11]. Infiltrating polymorphonuclear leukocytes, such as neutrophils, are the principal cellular components acting on the inflammatory response at the wound site [12, 13]. Reactive oxygen species (ROS) such as hydroxyl (**·**HO), superoxide anion radicals (O_2_^−^), and hydrogen peroxide (H_2_O_2_) are released by neutrophils to eliminate infectious threats [14, 15]. In the collagen-based stem cell delivery system, more neutrophils are supposed to accumulate in wound site along with the biodegradation process of collagen, as the generated peptide fragments of collagen were found as chemotactic factors and activators of neutrophils [16]. The activation of neutrophils results in several distinct morphological and metabolic events, all leading to phagocytosis and degradation of foreign substances to generate overabundant ROS [17–24]. Such overproduction of ROS cause an imbalance in the metabolism of reactive intermediates leading to oxidative stress damage in MSCs, and ultimately damage critical components of the implanted MSCs including their lipids, proteins, and DNA [25–32]. Hence, improving the ROS scavenging ability of the collagen-based scaffold to control the amount of ROS in microenvironment is of great concern, which will effectively avoid the hostile ROS-induced stem cells death, so as to preserve the essential paracrine factors secreted by stem cells to enhance tissue repairing capability.

Chitosan, a linear polymer of d-glucosamine with a β-(1, 4) linkage, with similar structure to the glycosaminoglycan portion of ECM, is a fascinating candidate for medical and pharmaceutical applications due to its unique biological properties [33–37]. It is completely biocompatible, bacteriostatic, hemostatic, and biodegradable of harmless substances and appears to act as a wound healing accelerator [38, 39].

**Scheme 1:**
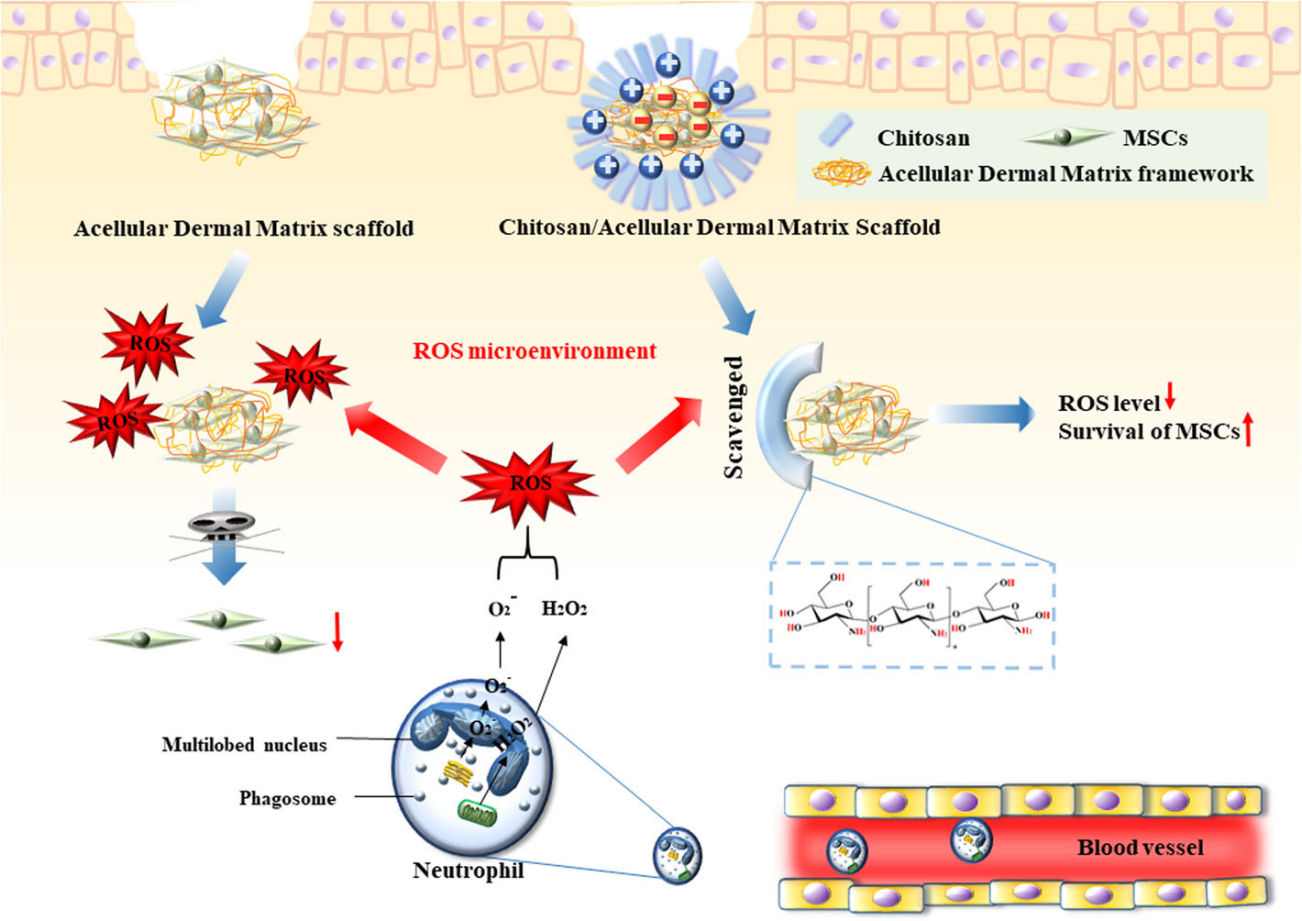
Effects of chitosan/acellular dermal matrix scaffold on the biological behaviors of MSCs in vivo during wound healing

Herein, we attempt to functionally modify ADM with chitosan to synthesize a novel chitosan/acellular dermal matrix (CHS/ADM) scaffold, which is applied to delivery MSCs for treating wound healing (Scheme 1). Chitosan is a polycationic material, while ADM is a type of polyanionic material that has a negatively charged surface, through the electrostatic interactions, chitosan will interact with ADM to form CHS/ADM. Under high ROS microenvironment which could be induced by collagen degradation in ADM, chitosan scavenges an amount of ROS and acts as an efficient barrier to protect MSCs from the oxidative stress. The ROS scavenging activity of chitosan are based on a hydrogen-donating ability of amine and hydroxyl group. The hydroxyl groups in the polysaccharide unit of the chitosan react with **·**OH by the typical H-abstraction reaction. In addition, the residual free amino groups NH_2_ in chitosan react with **·**OH to form stable macromolecule radicals [15, 40]. Chitosan is a copolymer of glucosamine and *N*-acetylglucosamine derived from the natural polymer chitin and could be metabolized in the body [41, 42]. Moreover, the degraded products of chitosan are nontoxic, nonimunogenic, and noncarcinogenic [43]. Therefore, this barrier against ROS would not have adverse reactions as it will ultimately degrade into harmless productions. This novel CHS/ADM stem cell delivery system is supposed to offer a favorable 3D matrix and reduce ROS-induced damage, which may significantly enhance the viable retention of implanted stem cells and further accelerate wound healing. To evaluate this, we first demonstrated the ROS scavenging capacity of the chitosan and then revealed the effectiveness of CHS/ADM scaffolds in decreasing the deterioration of MSCs survival by ROS. In particular, the effects of the scaffolds on neutrophils behavior and ROS release were assessed, which was followed by the investigation of viable retention of MSCs and therapeutic efficiency in an in vivo mouse excisional wound repair model.

## Method

### Materials

Chitosan (≥ 80% degree of deacetylation, M. W 40 w–70 w, YuanyeBio, Shanghai, China,), collagenase (BioFroxx, Guangzhou, China), and 2.5% glutaraldehyde water solution was purchased from Shanghai Pharm. Co. (China). 0.25% Dispase (Aoboxing, Beijing, China), cell counting kit-8 (CCK-8), C57BL/6 mouse bone marrow mesenchymal stem cells (GFP^+^-MSCs, MUCMX-01101, Cyagen Biosciences, Inc.), and C57BL/6 mouse bone marrow mesenchymal stem cells (MSCs, MUBMX-01001, Cyagen Biosciences, Inc.) were also used along with OriCell mouse MSC growth medium and adipogenic and osteogenic differentiation medium (Cyagen Biosciences, Guangzhou, China). Dulbecco’s modification of Eagle’s medium (DMEM) and 0.25% trypsin-ethylenediaminetetraacetic acid (EDTA) were acquired from Gibco (Grand Island, NY). Other reagents were obtained from China National Medicine Corporation and used as received. Milli-Q water was used in all experiments.

### Preparation and characterization of the CHS/ADM scaffold

ADM was prepared with 5-week-old BALB/c mouse skin as described previously [8]. Briefly, the mice were euthanized, and the hair was shaved. The full-thickness skin was then harvested from the bare mice. Next, the skin tissues were immersed in 0.25% dispase at 4 °C for 48 h to remove the epidermis. The processed skin tissues were then washed using PBS and immersed in 0.3% Triton X-100, 0.25% sodium deoxycholate, and 0.02% trypsin-EDTA with continuous shaking at 37 °C for another 48 h in a horizontal shaker to remove the cellular components from the matrix. The acellular scaffolds were treated with a fat digestion solution of methanol and chloroform (v/v = 1:1) for 2 h and washed with PBS. Finally, the scaffolds were cut into 8-mm-diameter round pieces for use. The prepared ADM was freeze-dried overnight firstly. Chitosan are positively charged in acidic medium, which was prepared as a 0.5% (w/v) solution by means of 0.5 M acetic acid solution. An ultrasonic cleaner was used to homogenize the chitosan solution. Further dilutions were necessary to obtain the various concentrations of stock solutions. The prepared ADM is initially denatured for 15 min at 60 °C water solution and then a solution of chitosan thermostated at 40 °C is added to the prepared ADM. Chitosan was then self-assembled on the ADM scaffold to produce CHS/ADM scaffolds. Next, the scaffold was neutralized with the 1 M NaOH aqueous solution for 2 h. Then, the samples were rinsed carefully with ultrapure water several times before lyophilization for 24 h to obtain the CHS/ADM scaffold. Simultaneously, the ADM scaffold was immersed in PBS as the comparison group (ADM scaffold). Fourier transform infrared spectroscopy (FT-IR) of chitosan, ADM and CHS/ADM scaffolds were recorded by a Nicolet iS50 spectrometer (Thermo Scientific Inc., USA). The biodegradation stability of the ADM and CHS/ADM scaffolds was analyzed by treatment with 10 mL of PBS containing 100 μg mL^− 1^ collagenase after weighing the initial weight (dry) of these scaffolds (*W*_0_). The biodegradation stability of ADM and CHS/ADM scaffolds in ROS environment was also studied with the same treatment. The degradation rates of these samples were explored at 24, 48, 72, and 96 h at the temperature of 37 °C, replacing the fresh collagenase solution every 12 h. The scaffolds were simultaneously removed from the degradation solution at a preset time point, carefully rinsed several times with deionized water, and freeze-dried after which the weight was recorded (*W*_t_). Finally, the following formula was used to calculate the degradation rate: DR% = (*W*_0_ − *W*_t_)/*W*_0_ × 100%. All experiments were performed in triplicate.

The ADM, CHS/ADM scaffolds, and the scaffolds after degradation for 24 h were then fixed in 2.5% glutaraldehyde at 4 °C for 24 h. Next, the scaffolds were dehydrated with graded ethanol (30%, 50%, 75%, 90%, and 100%) for 15 min and immersed in hexamethylene diamine (3 × 15 min). The dried scaffolds with a CO_2_ critical point were coated with a 30 nm gold layer for scanning electron microscopy (Zeiss Ultra 500; Carl Zeiss, Jena, Germany). Optimum SEM imaging parameters were as follows: extra high tension was set to 2 kV and the operating distance was 5.4 mm. The pore size and porosity distribution of the scaffolds were analyzed by Image-Pro Plus software.

### Cell culture

The MSCs and GFP^+^-MSCs were cultured in OriCellTM mouse MSC Growth Medium (MUCMX-90011, Cyagen Biosciences, Inc.), added by 10% fetal bovine serum (FBS, Gibco) and 1% penicillin–streptomycin in a humidified incubator (Thermo Scientific Forma 3110, Thermo Fisher Scientific, Inc.) at 37 °C, 5% CO_2_. The ADM and CHS/ADM scaffolds were exposed to UV light overnight before the experiment. Round pieces of ADM and CHS/ADM scaffolds (8 mm in diameter) were acquired via biopsy punches and placed in a 48-well plate (one piece per well) with the papillary dermis side facing upward. ADM and CHS/ADM scaffolds were incubated overnight in fresh MSC Growth Medium, and then 1 × 10^5^ MSCs were seeded on papillary dermis side of each ADM and CHS/ADM scaffolds and continued to culture in the MSC Growth Medium.

After 24 h, the GFP^+^-MSCs on the ADM and CHS/ADM scaffolds were photographed via a Carl Zeiss TPLSM 710 NLO META laser scanning microscope with a laser wavelength of 820 nm. A cell counting experiment was also performed to quantitatively analyze the viability of MSCs on ADM and CHS/ADM. Briefly, the MSCs growing on the scaffold were enzymatically digested and resuspended into a single cell suspension. Cell counting was performed in cell counting plate after trypan blue staining (*n* = 6).

### Adipogenic and osteogenic differentiation assays

After MSCs were cultured in 6-well dishes (control, chitosan, H_2_O_2,_ H_2_O_2_+chitosan) or on scaffolds (control, ADM, and CHS/ADM) for 1 day, the differentiation groups were treated with adipogenic and osteogenic medium for 14 and 21 days, respectively. Oil Red O staining and alizarin red staining were performed to verify adipocytes and osteoblasts, respectively. The morphologies of adipocytes and osteoblasts in the control (untreated), chitosan, H_2_O_2_, H_2_O_2_+chitosan groups were photographed and then counted under microscope for statistical analyses. Total mRNA from the ADM and CHS/ADM scaffolds was isolated for RT-PCR analysis. Each experiment observed three random fields per sample and was repeated three times.

### Measurement of cell viability, adhesion, and intracellular ROS

For the MSC viability assay, hydrogen peroxide (H_2_O_2_) was used as the exogenous ROS source, and MSCs were treated with different concentrations of H_2_O_2_ and detected by the CCK-8 assay. The MSCs treated with different chitosan concentrations were also detected by the CCK-8 assay to investigate MSC viability in the ROS environment. Each sample was treated with 400 μL of serum-free L-DMEM medium plus 40 μL of CCK-8 solution per well. After 6 h of incubation at 37 °C, the resulting production of water-soluble formazan dye was assayed at a wavelength of 450 nm by an EnspireTM multimode reader. Each sample was repeated three times in parallel. Simultaneously, calcein-AM/propidium iodide (Calcein-AM/PI) staining was used to evaluate the MSCs viability treated with different chitosan concentrations under ROS microenvironment. The excitation/emission wavelength of AM is 480 nm/452 nm. The excitation/emission wavelength of PI is 535 nm/617 nm. For MSC-matrix adhesion assay, viable MSCs were suspended in medium to prepare cell suspensions and then were added to each well of a six-well plate and allowed to attach for 2 h at 37 °C and 5% CO_2_. Chitosan was used as ROS scavengers to evaluate their protection of MSCs from ROS. After adhesion, plates were carefully washed three times with PBS, and then five separate fields were counted under microscope. Each experiment was repeated at least three times. Subsequently, the intracellular ROS of the MSCs with the treatment of chitosan under the ROS environment were measured by immunofluorescence assay with dihydroethidium (DHE) staining.

MSCs (1 × 10^5^) were cultured for 24 h and then seeded in CHS/ADM and ADM scaffolds for 24 h (ADM scaffolds are as the comparison group). Calcein-AM/PI staining was used to evaluate the MSC survival activity seeded on scaffolds under ROS microenvironment. The CCK-8 assay was also performed to investigate MSC viability (%) seeded on scaffolds in the ROS environment. In addition, the intracellular ROS of the MSCs-ADM and MSCs-CHS/ADM scaffolds under the ROS environment were measured. The fluorescence intensity was analyzed using ImageJ software. Each experiment was repeated at least three times.

### In vivo studies

#### Mouse model and implantation of MSCs seeded on the scaffold

The specific pathogen-free BALB/c mice (7–8 weeks) were supplied by the animal facility of Southern Medical University. All animal procedures were conducted in strict accordance with the Institutional Animal Care and Use Committee of the University of South China Normal University. The dorsal skin of each mouse was shaved and sterilized with 70% ethanol and iodine prior to surgery. Animals were anesthetized with an intraperitoneal injection of chloral hydrate (400 mg/kg, Fuchen, Tianjin, China). Once the mice were anesthetized, an 8-mm biopsy punch was used to make an impression on the dorsum, and then the circular region of tissue was grabbed and pulled with forceps and excised with scissors to create a full-thickness wound. The wounded mice were randomly subjected to three groups (*n* = 6), which were separately treated as control (untreated wound), MSCs-ADM scaffold or MSCs-CHS/ADM scaffold. Checks for postoperative pain and complications were performed daily after the operation.

#### Neutrophil infiltration and intracellular ROS

The cutaneous wound repair areas were dissected after treatment with the MSCs-ADM and MSCs-CHS/ADM scaffolds after 1, 3, and 5 days. Then, the samples were inserted into paraffin and were perpendicularly sectioned into 3-μm-thick longitudinal sections. The paraffin sections were stained with CD11b after deparaffinization and rehydration, and then washed with ultrapure water for histological analysis.

For immunofluorescence staining with dihydroethidium (DHE, Servicebio, Guangzhou, China), the frozen sections were slightly dried, a pap pen was used to make a circle around the tissue, and DHE was added to the circle followed by incubation at 37 °C for 30 min in the dark. After washing with PBS, all slices were stained with 4′,6-diamidino-2-phenylindole (DAPI, 5 μg mL^− 1^, Sigma) to reveal the nuclei. Images were acquired by a confocal laser scanning microscope. Relative fluorescence intensity was analyzed with ImageJ software.

#### Two-photon excitation fluorescence (TPEF) and second harmonic generation (SHG) imaging of the MSCs-CHS/ADM scaffold transplanted into the wound site

Mice were anesthetized and disinfected with alcohol to dissect the cutaneous wound repair areas after treatment with the MSCs-ADM and MSCs-CHS/ADM scaffolds after 7, 14, 21, and 28 days. Finally, the samples were flattened and placed on clean glass slides. These tissue samples were imaged by two photon laser scanning confocal microscope (TPLSCM). Collagen in ADM or CHS/ADM scaffold was imaged by SHG signals with the emission wavelength of 405–415 nm (central wavelength of 410 nm) at a laser excitation wavelength of 820 nm. The regenerated wound areas in each sample were continuously scanned (a frame of a 2D image) for collagen and green fluorescence protein (GFP) simultaneously at a speed of 6 s per frame. To identify whether the selected tissue samples were viable, the exposure time of the samples was strictly limited to within 30 min. The intensities of the GFP and SHG signals were analyzed using ImageJ software.

#### Real-time quantitative PCR analysis

Total RNA from the wound samples and single cell suspensions was extracted with an RNA extraction kit (AIVD, Guangzhou, China). Then, cDNA synthesis was performed using a PrimeScript™ RT reagent Kit with gDNA Eraser (TaKaRa, Dalian, China). The RT-PCR primer sequences were synthesized by Sangon Biotech (Shanghai, China) in Table 1:

**Table 1:** Primer sequences

Gene Forward primer (5′–> 3′)	Reverse primer (5′–> 3′)
β-actin CGTTGACATCCGTAAAGACC	TAGGAGCCAGAGCAGTAATC
GFP GCACGACTTCTTCAAGTCCGCCATGCC	GCGGATCTTGAAGTTCACCTTGATGCC
LPL AGTTTGACCGCCTTCCGCGG	TCCTGTCACCGTCCATCCATGGA
PPARγ ACTGCCGGATCCACAAAA	TCTCCTTCTCGGCCTGTG
ALP AACCCAGACACAAGCATTCC	CCAGCAAGAAGAAGCCTTTTG
Runx-2 TGCCACCTCTGACTTCTGCC	CGCTCCGGCCCACAATCTC

PCR was conducted on a TaKaRa Real-Time PCR machine using SYBR Green (SYBR® Premix Ex Taq™, TaKaRa, Beijing, China) and validated with a CFX96 Real-Time PCR system (Applied Biosystems). The mRNA expression level of the target gene was normalized to β-actin expression and was analyzed using the comparative method of relative quantification (2^−ΔΔ^Ct). Five repetitions were performed on each sample.

### Western blotting

Protein lysates from the wound samples were homogenized in RIPA buffer containing protease inhibitors and denatured at 95 °C in sample buffer for 5–10 min. Each sample protein was electrophoresed on 12% SDS-PAGE and then transferred to PVDF membranes. The membranes were blocked in buffer (5% nonfat dry milk, 10 mM Tris, 100 mM NaCl, 0.1% Tween-20) at 4 °C overnight and incubated with rabbit polyclonal GFP antibody (1:1500 dilution, Abcam) and mouse monoclonal β-actin antibody (1:2000 dilution, Affinity) at 4 °C overnight. After washing, the membranes were incubated with secondary antibodies at room temperature for 2 h and washed with TBST three times for 10 min each time. The detection of the western blot signals was performed with an ODYSSEY Infrared Imaging System (LI-COR) and the generated signals were analyzed using ImageJ software.

### Immunofluorescence and histological analysis

Mice from each group were photographed on different days after different wounding days (7, 14, 21, and 28). The percent wound closure was calculated by Image-Pro Plus software and quantified with the follow equation:

Wound closure rate (%) = (initial wound area − indicated wound area)/initial wound area × 100%.

In addition, paraffin sections of wound tissue were autoclaved for antigen retrieval and blocked with a peroxide blocking agent and normal goat serum (Beyotime, Guangzhou, China). The samples were incubated with primary antibodies to green fluorescence protein (GFP, 1:1000, Sigma) and vascular smooth muscle actin (α-SMA, 1:1000, Millipore Chemicon) for double-labeled immunofluorescence staining, as well as α-SMA for single-labeled immunofluorescence staining. After washing, the samples were incubated with the following secondary antibodies: CY3-labeled goat anti-rabbit IgG (for GFP, 1:300, Abcam) and FITC-labeled goat anti-rat IgG (for α-SMA, 1:300, Abcam). All slices were stained with 4′,6-diamidino-2-phenylindole (DAPI, Sigma) to show the nuclei. Images were acquired by confocal laser scanning microscopy. Masson’s trichome staining was used to analyze the regeneration of the collagen accumulation state with ImageJ. In addition, hematoxylin and eosin (H&E) staining was carried out for the histological analysis of wound regeneration. All data were analyzed using ImageJ software.

### Statistics analysis

All statistical results are presented as the means ± SD. Statistical analyses were performed with OriginPro 8.5 (OriginLab, USA), and significant differences were assessed with one-way ANOVA and Dunnett’s multiple comparison tests, with statistical significance levels denoted by a single asterisk (*p* < 0.05), two asterisks (*p* < 0.01), or three asterisks (*p* < 0.001), and are indicated in each figure.

## Results and discussion

### Chitosan enhances the survival activity of MSCs under ROS microenvironment

**Fig. 1:**
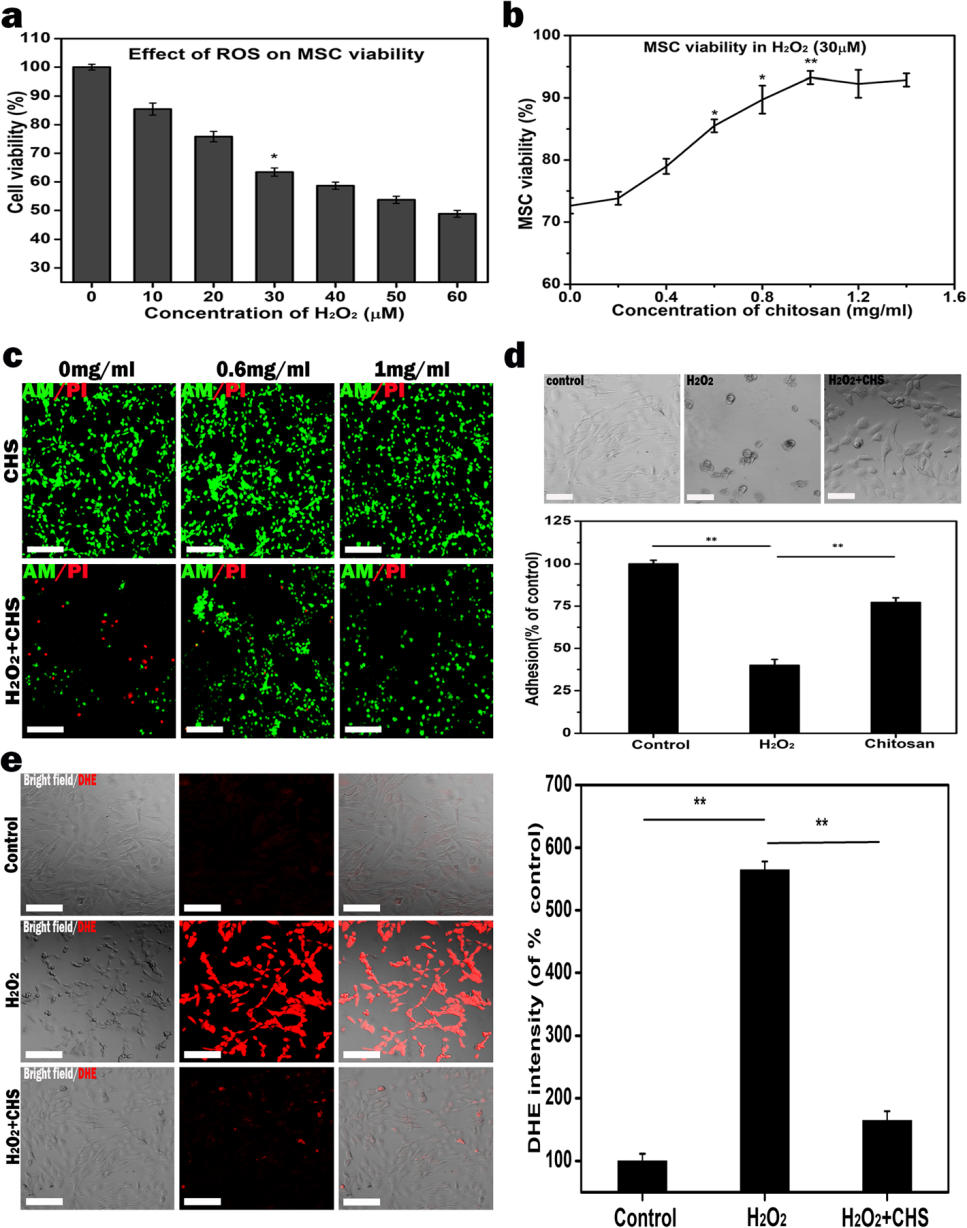
ROS scavenging capacity of the chitosan. **a** ROS inhibits MSC viability in a dose-dependent manner. **b** Various chitosan concentrations restore the reduced viability of MSCs in a dose-dependent manner. **c** Live/dead staining images of the MSCs with H_2_O_2_ treatment. Green and red represent lived cells and dead cells, respectively. Scale bars are 200 μm (*n* = 5). **d** Images of adhesive cells on culture plastic surface in presence of H_2_O_2_. Quantification of adhesive MSCs shows that ROS significantly inhibits the adhesion of MSCs compared to control (untreated with H_2_O_2_), while chitosan significantly attenuates the effect induced by ROS. **e** The measurement of intracellular ROS by DHE staining. Control indicates untreated with H_2_O_2_, H_2_O_2_ indicates H_2_O_2_ treatment, and H_2_O_2_+CHS indicates H_2_O_2_+chitosan treatment; scale bars are 200 μm; **p < 0.01, *p < 0.05

An in vitro coculture was performed to evaluate the survival ability of MSCs with chitosan under ROS microenvironment. Hydrogen peroxide (H_2_O_2_) was used to imitate ROS environment that could lead to induce oxidative stress damage in MSCs. As shown in Fig. 1a, MSC viability was inhibited by H_2_O_2_ in a dose-dependent manner compared with the control. These data reveal that ROS inhibits MSC viability and results in MSC death. Once the concentration of H_2_O_2_ was above 30 μM, the survival of MSCs was apparently reduced; therefore, 30-μM concentration was selected and applied in the following detriment investigation of H_2_O_2_. Next, the antioxidant activity of the chitosan on MSC viability under H_2_O_2_-treated (30 μM) was investigated and the H_2_O_2_-untreated was used as the control group. The introduction of chitosan recovered the ROS-reduced MSC survival rate in a dose-dependent manner, especially when the chitosan concentration was 1 mg/ml (Fig. 1b, c). Likewise, in the assay of cell-matrix adhesion, while the adhesive MSCs were reduced to 37.8 ± 1.37% of control by H_2_O_2_ treatment (30 μM) (**p < 0.01), the addition of 1 mg/ml chitosan could recover the reduced MSC adhesion induced by ROS (Fig. 1d). Also, as Fig. 1e demonstrates, the incorporation of 1 mg/ml chitosan significantly decreases the level of intracellular ROS in H_2_O_2_ environment. Therefore, the CHS/ADM scaffold with the 1 mg/ml concentration of the chitosan was chosen for the next experiment.

The effect of chitosan on differentiation capability of MSCs is then to be explored, which is crucial because it is one of the most significant characteristics of MSCs for their application in regenerative medicine. By staining adipocytes and osteoblasts with Oil Red O and Alizarin red, respectively, chitosan was confirmed to have no significant influence on differentiation of MSCs (Fig. S1a). Further experiments showed that H_2_O_2_ decreased the adipogenic and/or osteogenic differentiation, while chitosan could effectively prevent the H_2_O_2_-induced decrease (Fig. S1b).

### The characterization of the CHS/ADM scaffold and its effect on the survival and differentiation of MSCs

**Fig. 2:**
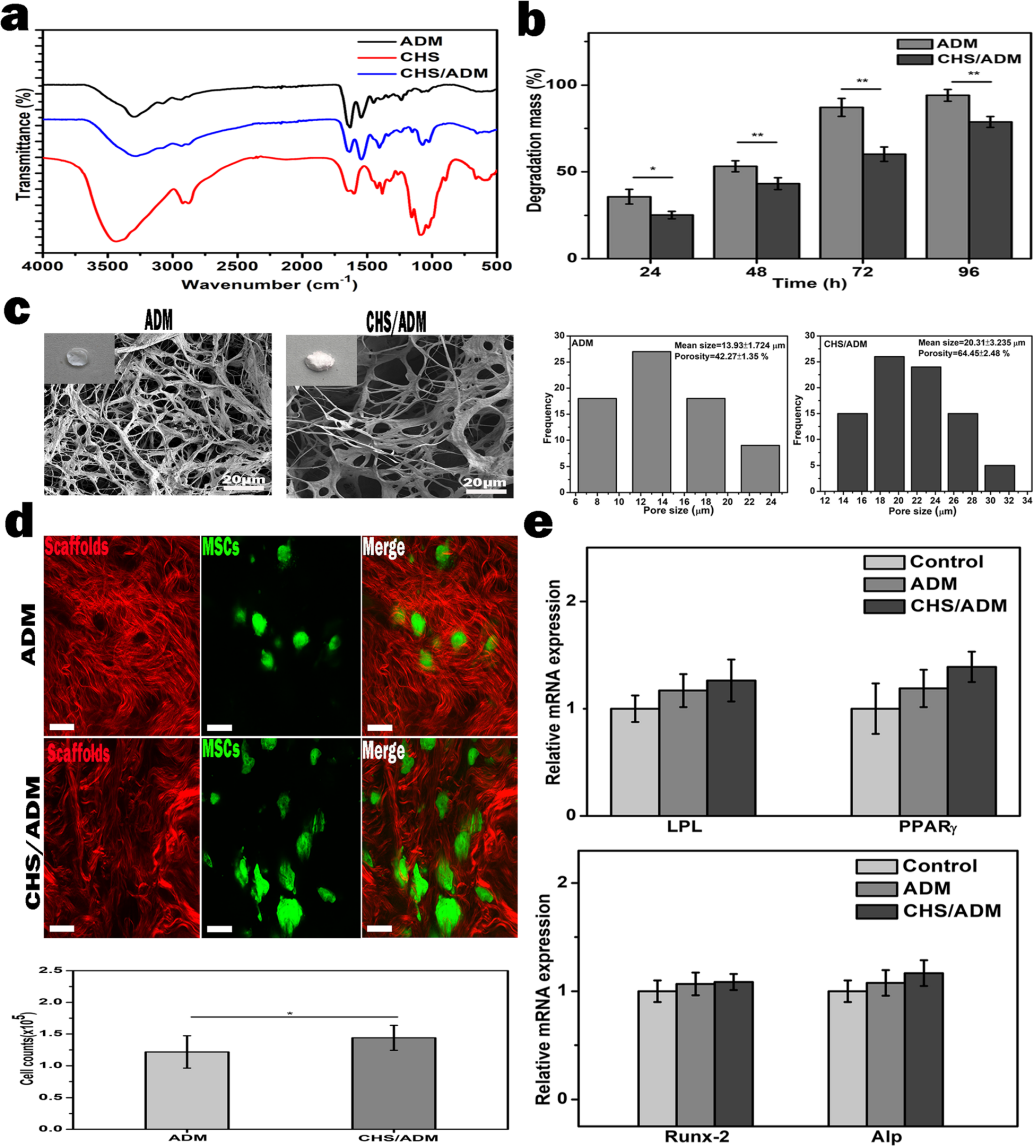
The characterization of the CHS/ADM scaffold and its effect on the survival and differentiation of MSCs. **a** FT-IR absorption spectra of the CHS, ADM, and CHS/ADM scaffolds. **b** The percent degradation mass of the ADM and CHS/ADM scaffolds after enzymatic degradation at different time points (**p < 0.01, *p < 0.05, *n* = 3). **c** SEM images showing the morphologies of the ADM and CHS/ADM scaffolds. The corresponding illustrations show 8-mm-diameter ADM and CHS/ADM scaffolds. Scale bar, 20 μm. The corresponding pore size and porosity distribution of the ADM and CHS/ADM scaffolds are shown right (*n* = 3). **d** TPEF-SHG imaging and cell counting of MSCs cultured on the ADM and CHS/ADM scaffolds for 24 h (*n* = 3). Scale bar, 10 μm. **e** Quantitative analysis of the mRNA expression levels of specific adipocyte genes (LPL, PPARγ) and osteogenic genes (Runx-2, ALP) of MSCs seeded on either the ADM and CHS/ADM scaffolds or only in medium as the control on days 14 and 21

FT-IR spectra from ADM, chitosan (CHS), and CHS/ADM are shown in Fig. 2a. The spectra of ADM demonstrate four characteristic absorption bands at 3321, 1640, 1553, and 1238 cm^− 1^. Generally, amide I bands (1640 cm^− 1^) originate from C=O stretching vibrations coupled to N-H bending vibrations. The amide II bands (1553 cm^− 1^) arise from the N-H bending vibrations coupled to C–N stretching vibrations. The amide III band (1238 cm^− 1^) is the combination peak between N-H deformation and C-N stretching vibrations. The vibrations of hydroxyl group, −OH, appear at 3306 cm^− 1^ [44]. The spectra of chitosan contain six characteristic absorption bands. The vibrations of −OH and free −NH_2_ appear at 3455 and 3364 cm^− 1^, respectively. The absorption bands at 1648, 1592, and 1381 cm^− 1^ are amide I, amide II, and NH_2_ bending and C–O stretching of primary alcohol groups, respectively. The last one, at 1098 cm^− 1^, represents C–O–C glycosidic linkage between chitosan monomers [45–47]. FT-IR spectra of CHS/ADM illustrate similar characteristic peaks of the parent molecules, hence indicating that collagen and chitosan interactions are polyelectrolytic with oppositely charged ionic polymers, particularly the cationic group of chitosan (−NH_3_^+^) and negative group in anionic collagen (−COO^−^) [47–49]. The degradation degree of the ADM and CHS/ADM scaffolds is also shown in Fig. 2b. The results illustrate that both scaffolds displayed an increasing enzymatic degradation with time increasing. After 96 h, the degradation mass of the ADM scaffold was approximately 94.12 ± 3.37%, but the CHS/ADM scaffolds had a lower degradation rate (78.75 ± 3.17%), which indicates that the degradation rate of ADM scaffold becomes slower with the introduction of chitosan. The lower degradation rate in CHS/ADM scaffold is not exactly known, but it could be that the chitosan blocks collagenase binding sites in collagen and/or directly inhibits enzymatic activity of collagenase, thereby enhancing the resistance to degradation of collagen [50].

As shown in Fig. 2c-inset, The CHS/ADM scaffolds reveal looser than the ADM scaffolds. This was also demonstrated from the scanning electron microscopy images in Fig. 2c. The results show that the ADM scaffold comprised a collagen fiber network structure with a limited pore size (13.93 ± 1.724 μm) and porosity (42.27 ± 1.35%). In contrast, the CHS/ADM scaffold exhibited a network structure of chitosan-coupled collagen fiber with an optimized pore size (20.31 ± 3.235 μm) and porosity (64.45 ± 2.48%) (Fig. 2c, right). This bigger pore size in CHS/ADM scaffold is possibly mainly caused by rehydration and relyophilization process in the refreeze-drying treatment in the preparation of the CHS/ADM scaffold. This refreeze-drying procedure may lead to some collagen fibers in the ADM scaffold being combined with each other to form a sheet-like structure. As a result, the pore size is increased correspondingly [51, 52]. In addition, the content and the concentration of chitosan may improve the structural stability of the matrices in CHS/ADM scaffold which is related to the porosity [53, 54]. This bigger pore size and higher porosity might improve the binding capacity of the MSC [54].

Next, two-photon excitation fluorescence (TPEF) and second harmonic generation (SHG) imaging was performed to observe the morphological characteristics of GFP^+^-MSCs seeded on the scaffolds in real time (Fig. 2d). As expected, the MSCs seeded on the CHS/ADM scaffold showed more survival activity than that in ADM scaffold. Quantitatively, MSCs after 1-day seeding on the ADM and CHS/ADM scaffolds were counted by the cell counting plate after trypan blue staining. As shown in Fig. 2d, the number of the MSCs in CHS/ADM scaffold group is more than that in ADM scaffold, which indicates that CHS/ADM scaffold has desirable properties for MSCs survival. Additionally, the differentiation capability of the MSCs seeded on the scaffolds was explored; there was no significant difference in both adipocyte and osteoblast differentiation induction between CHS/ADM scaffold and those in ADM scaffold in Fig. 2e. The above data reveal that the CHS/ADM scaffold has appropriate structural characteristics and excellent biocompatibility, which is favorable as a potential candidate scaffold for long-term stem cell therapy.

### CHS/ADM scaffold enhances the survival activity of MSCs under ROS microenvironment

**Fig. 3:**
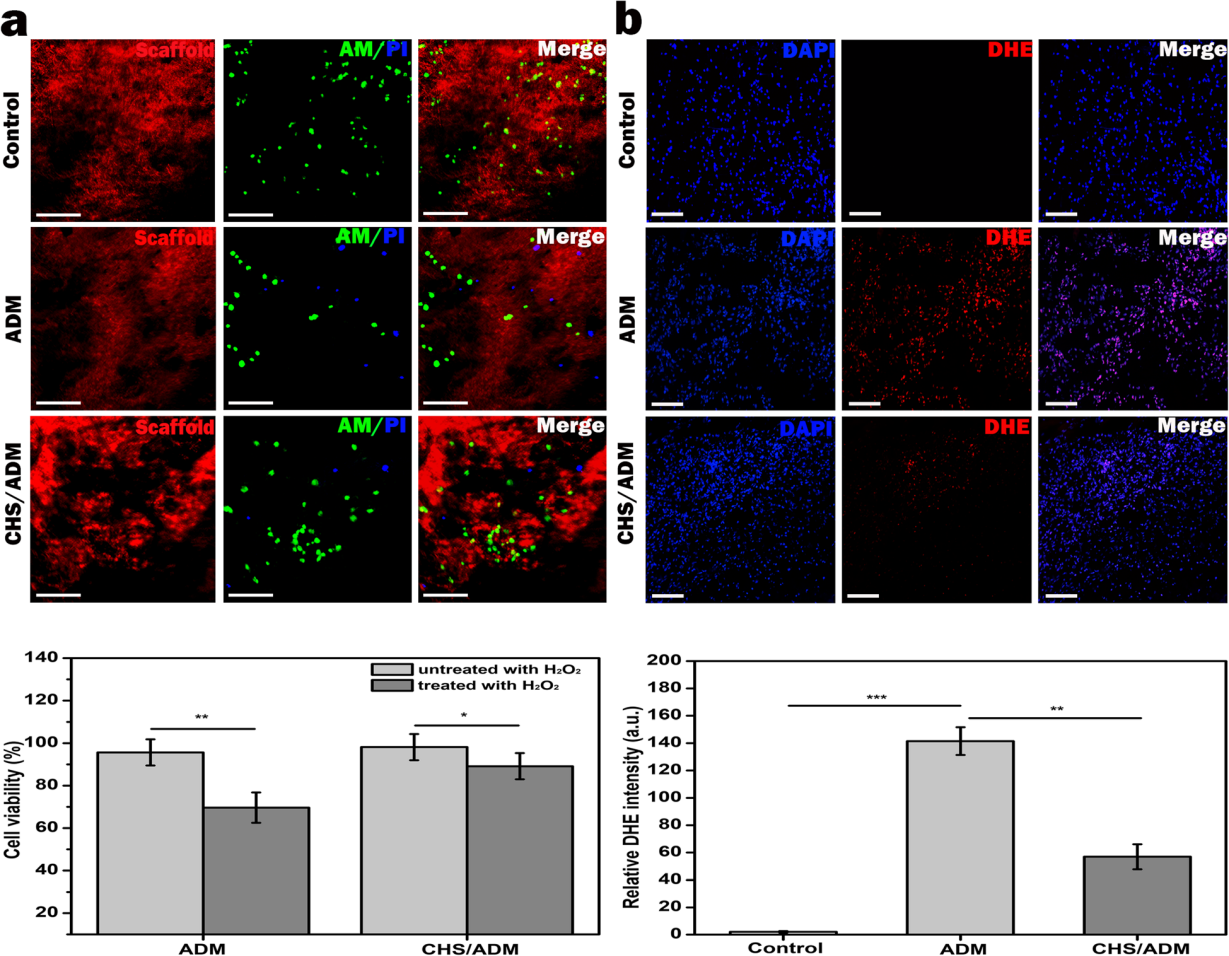
Cell protective capacity of the CHS/ADM scaffold under ROS microenvironment. **a** Live/dead staining images of the MSCs-ADM and MSCs-CHS/ADM scaffolds with H_2_O_2_ treatment (above). Control is MSCs-ADM scaffold untreated with H_2_O_2_. Green and blue represent lived cells and dead cells, respectively. Red represents SHG imaging of scaffolds. Scale bars are 200 μm (*n* = 3). Cell viability (%) of the MSCs-ADM and MSCs-CHS/ADM scaffolds treated with H_2_O_2_ or untreated with H_2_O_2_ (below). **b** Dihydroethidium (DHE) staining images of intracellular ROS in MSCs seeded on the ADM and CHS/ADM scaffolds with H_2_O_2_ treatment. Control is MSCs-ADM scaffold untreated with H_2_O_2._ Red and blue represent ROS and nuclei, respectively. The quantitative analysis of the relative DHE intensity (*n* = 5) is in below. Scale bars are 200 μm; ***p < 0.001, **p < 0.01, *p < 0.05

In Fig. 3a (above), the results of the live/dead assay showed that most cells were stained green, and only a few dead cells (blue) could be observed in the CHS/ADM scaffold after H_2_O_2_ treatment. And the MSC viability (%) by CCK8 assay shown in Fig. 3a (below) in the CHS/ADM scaffold is also better than that in ADM scaffold after H_2_O_2_ treatment. These data indicate that the CHS/ADM scaffold could improve the survival of MSCs in the ROS microenvironment compared with the ADM scaffold group. Furthermore, dihydroethidium (DHE) staining was performed to observe the level of intracellular ROS in MSCs-ADM or MSCs-CHS/ADM scaffolds in Fig. 3b. The results revealed that the level of intracellular ROS in ADM scaffold group significantly increased in H_2_O_2_ environment. In comparison, this increase in ROS was partially eliminated by the CHS/ADM scaffolds. These data revealed that the detrimental effects displayed by ROS could be evidently attenuated by the introduction of chitosan into the ADM scaffold via the scavenging of ROS. Most importantly, the levels of ROS are markedly lower in the CHS/ADM scaffold compared to the ADM scaffold, which further reduces MSC death caused by oxidative damage and strengthens the survival capacity of MSCs in the ROS microenvironment during wound healing.

### The CHS/ADM scaffold attenuates the inflammatory response and intracellular ROS at the wound site

**Fig. 4:**
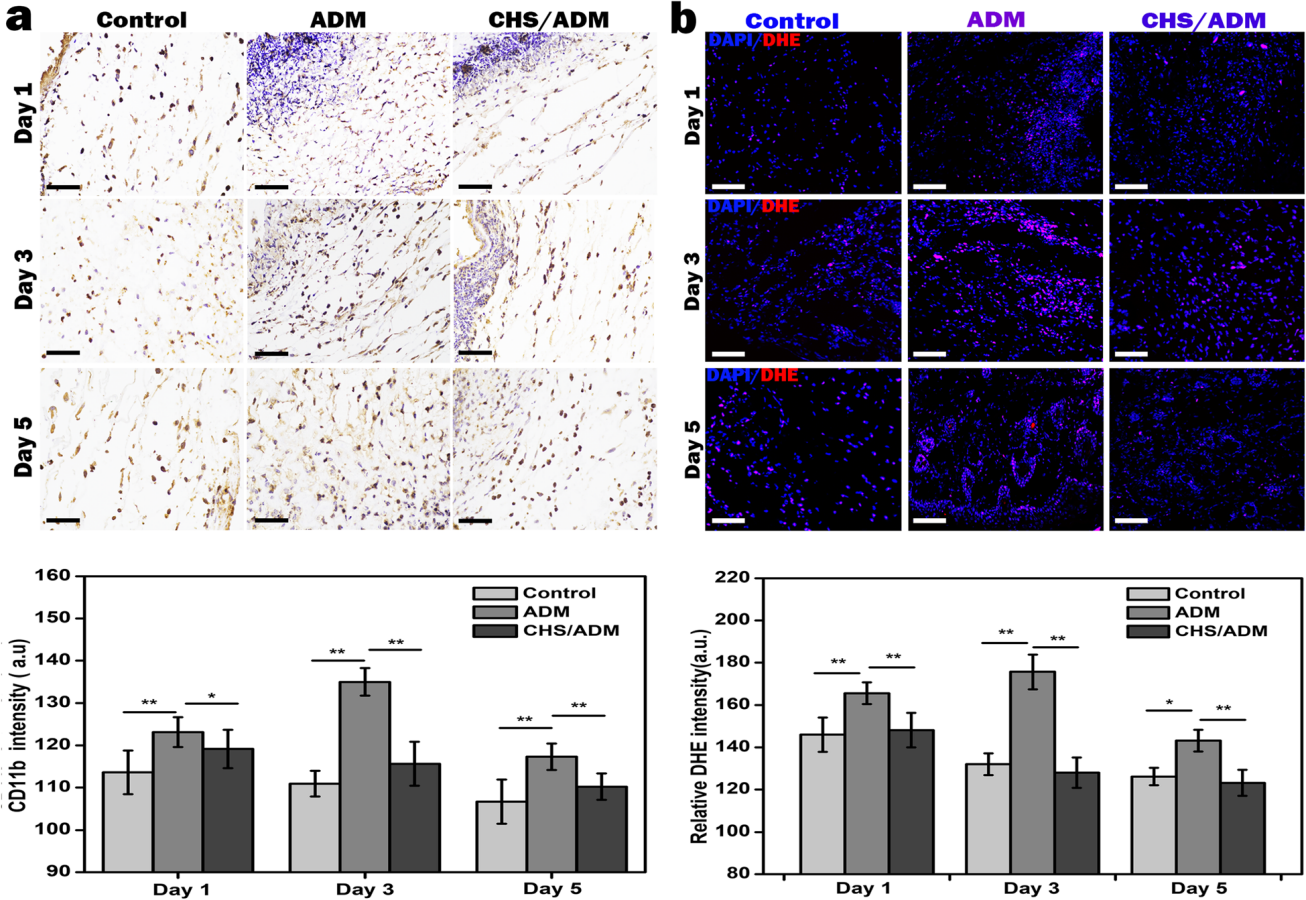
CHS/ADM scaffolds transplanted at the wound site during the inflammation period. **a** Immunohistochemical labeled with CD11b and the intensities analysis of the MSCs-ADM and MSCs-CHS/ADM scaffolds compared to the control (untreated wound) (*n* = 6). × 400, scale bar, 50 μm; **p < 0.01, *p < 0.05. **b** The measurement of intracellular ROS of the MSCs-ADM or MSCs-CHS/ADM scaffolds group compared to the control (untreated wound) by DHE staining on day 1, day 3, and day 5 in mice. And the quantification of the relative DHE intensity (*n* = 6). Scale bar, 200 μm; **p < 0.01, *p < 0.05

Neutrophils are a central component of the inflammatory response, which have the potential to cause severe tissue destruction by releasing an excess ROS onto host tissues [16, 55, 56]. It is essential to evaluate the effects of CHS/ADM scaffold on neutrophil and particularly focus on the amount of ROS. As inflammatory response usually occurs at the first few days of injury and neutrophils are short lived, they may disintegrate and disappear after 2 or 3 days. Therefore, in order to understand the neutrophil infiltration and ROS production level during inflammatory response, the first days like day 1, 3, and 5 after injury were chosen as the time points for investigation. In the present study, immunohistochemical analysis of sections was performed by labeling with CD11b to identify the infiltrated neutrophils during the inflammation period. On day 1 after the operation, neutrophils were present in a moderate amount in the CHS/ADM scaffold group and subsequently decreased over time (Fig. 4a). However, significant amounts of neutrophils were observed in the ADM scaffold group during this period. As such, the CD11b intensity in the ADM scaffold group was obviously higher than that in the CHS/ADM scaffold group during the same period (*p < 0.05). These data demonstrate that the CHS/ADM scaffold alleviated the inflammatory response, which is beneficial for providing a favorable survival environment for implanted stem cells. The higher amount of infiltrated neutrophils in ADM scaffold are most likely due to collagen degradation, which is initially induced by recruited neutrophils because of injury to vascularized connective tissue through releasing collagenase [18]. As collagen degradation-derived products might directly serve as chemotactic stimuli for neutrophils in vivo [16, 57], the peptide fragments of collagen produced by the degradation of the ADM scaffold consequently lead to excessive neutrophil infiltration.

Simultaneously, immunofluorescence with dihydroethidium (DHE) staining was performed to explore the intracellular ROS in the MSCs seeded on scaffolds after transplantation at the wound site. In Fig. 4b, strong red fluorescence can be observed around/in the cell nucleus (blue) in ADM scaffold at day 3, revealing more production of ROS compared to the control group. This overproduction of ROS in ADM scaffold is likely closely associated with the higher amount of infiltrated neutrophils in inflammation, as a high concentration of ROS is confirmed usually being released by neutrophils to stunt the growth of adjacent bacteria in wound environment [58]. Also, there is evidence that a continuous recruitment of inflammatory cells serves as a substantial source of ROS [59]. However, the CHS/ADM scaffold played an effective ROS scavenging role that contributed to a reduction in the red fluorescence during the same period. Moreover, the relative DHE intensity in the CHS/ADM scaffold group was apparently lower than that in the ADM scaffold group after 3 days (**p < 0.01), which is probably due to the introduction of chitosan in the composite scaffold that reduces ROS in the environment. These data show that the CHS/ADM scaffold attenuates the inflammatory response and reduces the amount of ROS in the environment.

### The CHS/ADM scaffold enhances MSC retention in wound healing

**Fig. 5:**
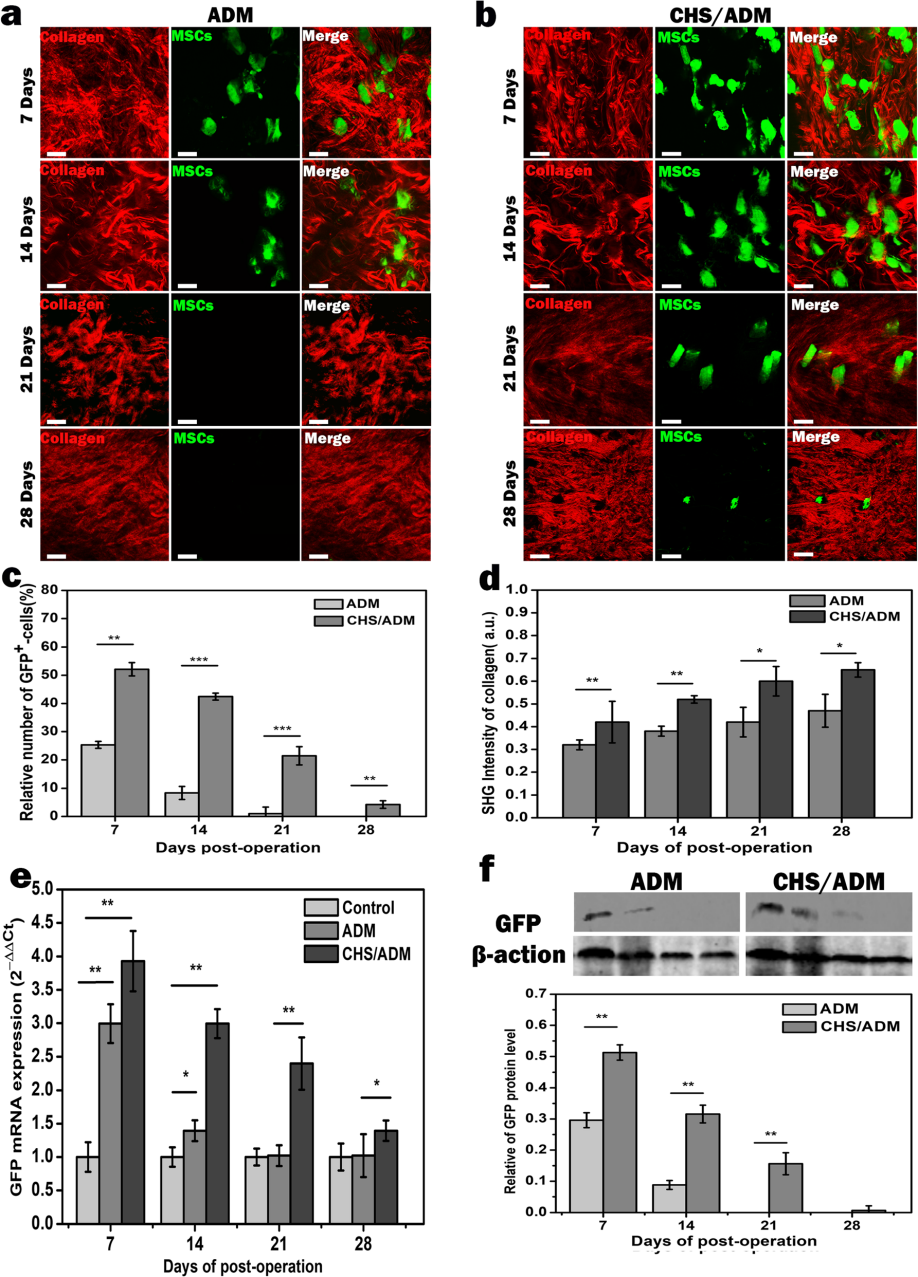
The CHS/ADM scaffold enhanced MSC retention in vivo. **b** TPEF-SHG imaging of MSCs that seeded on the CHS/ADM scaffolds transplanted into the wound site on days 7, 14, 21, and 28. Green and red represent GFP^+^-MSCs and collagen, respectively. **a** ADM scaffold as the comparison group (original magnification, × 60; scale bar, 10 μm). **c** The change in the number of MSCs in the wounds on days 7, 14, 21, and 28 after transplantation. **d** SHG intensity of collagen at different time points with increased treatment in the ADM and CHS/ADM scaffold groups. **e** Comparison of GFP levels in each group (7, 14, 21, and 28 days) via PCR and **f** western blot analysis. Statistical analysis of the expression of GFP. Data are expressed as the mean values, and the error bars represent the SD (*n* = 5). ***p < 0.001, **p < 0.01, *p < 0.05

Retention is the duration of survival. To reveal the retention of stem cells implicates determining their time of death. In order to analyze the ability of the CHS/ADM scaffold in improving implanted stem cell retention in wound repair, TPEF-SHG imaging was therefore performed on later days 7, 14, 21, and 28 after the transplantation of the MSCs-CHS/ADM scaffolds into skin wounds. At the same time, MSCs-ADM scaffolds were used as the comparison group. As shown in Fig. 5a and b, the amount of GFP^+^-MSCs in the CHS/ADM scaffold group was significantly higher than that in the ADM scaffold group at the same time period. Moreover, a dramatic decrease in cell viability (green) was observed on day 14 after the operation in the ADM scaffold group. Through evaluation of the GFP and SHG signal intensities, the changes in the number of GFP^+^-MSCs and collagen accumulation were depicted at different time points, as shown in Fig. 5c and d. In addition, as the treatment time increased, the newborn collagen content of both scaffolds obviously increased, and the collagen content of the CHS/ADM scaffold group was higher than that of the ADM scaffold group. It is worth noting that cells delivered in CHS/ADM scaffolds displayed markedly more GFP^+^-MSCs than that of ADM scaffolds on the same healing day, and GFP^+^-MSCs retained till day 28 in the CHS/ADM scaffold group, while under ADM scaffold, GFP^+^-MSCs disappeared after 14 days of implantation. These results were further confirmed by PCR and western blot analyses (Fig. 5e, f). The mRNA and protein expression levels of GFP in the CHS/ADM scaffold group were markedly higher than those in the ADM scaffold group during the same time period. The level of GFP decreased in the wounds over time and downregulated to zero till day 28, which is much longer than the day in ADM group (about 14 days). These results suggest that the CHS/ADM scaffold delivery system effectively enhanced MSC retention in the hostile wound microenvironment over 3 weeks, which is advisable for wound healing.

### Evaluation of angiogenesis response

**Fig. 6:**
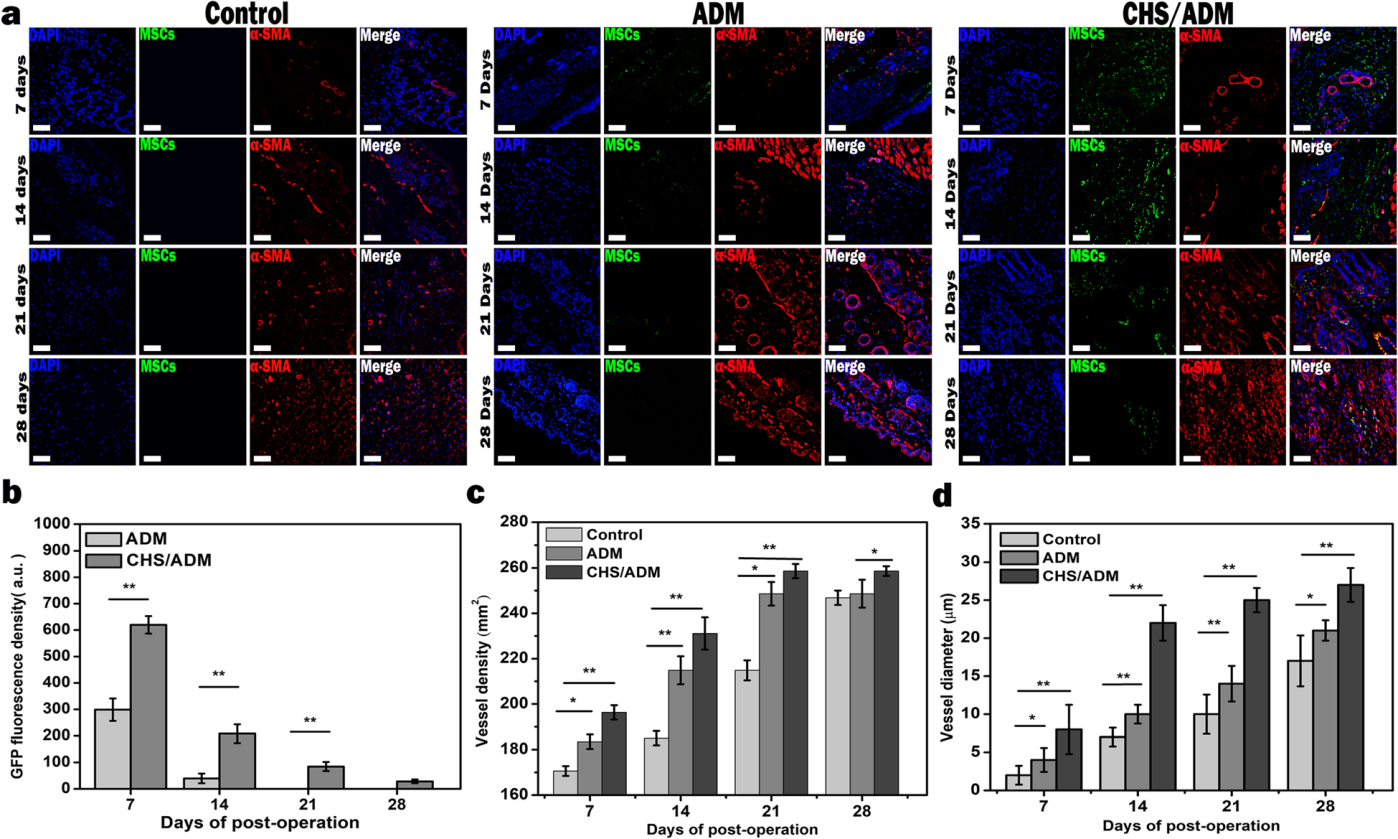
The occurrence of vascular regeneration at the wound site. **a** Fluorescence imaging of wound tissues in the control, MSCs-ADM scaffold, and MSCs-CHS/ADM scaffold groups on days 7, 14, 21, and 28. Green, red, and blue represent GFP^+^-cells, α-SMA^+^-cells, and nuclei, respectively. Scale bar, 200 μm. **b** Fluorescence intensities of GFP with different treatment times. Quantitative analysis of **c ** the vessel density and **d** diameter of the MSCs-ADM scaffold, MSCs-CHS/ADM scaffold and control groups on days 7, 14, 21, and 28. Data are expressed as the mean values and SD (*n* = 5). **p < 0.01, *p < 0.05

Angiogenesis at the later stage of wound healing is generally used to evaluate the effect of wound healing. Angiogenesis was evaluated in sections of regenerated tissue at the wound sites by double-immunofluorescence staining with GFP and alpha-smooth muscle actin (α-SMA). The control group was only stained with α-SMA antibody (Fig. 6a). As the angiogenesis usually occurs later on 7 days, therefore, the later days like days 7, 14, 21, and 28 in wound healing were chosen as the time points for investigation. The results show that the wounds in the CHS/ADM scaffold group appeared to have substantially more GFP^+^-MSCs and α-SMA fluorescence than the untreated control and ADM scaffold groups at the same treatment day (Fig. 6a–c). The survival of the implanted GFP^+^-MSCs was quantified by the fluorescence intensity of the cells in the assay (Fig. 6b). The results further demonstrate that the retention time of GFP^+^-MSCs in the CHS/ADM scaffold group was extended, and few MSCs were detected on day 28.

In addition, alpha-smooth muscle actin (α-SMA) staining of wound sections from different treatment groups were performed to identify the vasculature with smooth muscle cells. Angiogenesis networks mainly appeared on day 14 in the CHS/ADM scaffold group, and annular mature vessels principally appeared after 21 days. Quantitative analysis indicated that the blood vessel density of the CHS/ADM scaffold group (231.08 ± 46 mm^2^) was significantly higher than that of the ADM scaffold group (214.87 ± 21 mm^2^) and control group (185.05 ± 25 mm^2^) (*p < 0.05) (Fig. 6c). Furthermore, the vessel diameter of the CHS/ADM scaffold group was larger than that of the ADM and control groups in the healing tissues (23.54 ± 4.17 mm, 13.54 ± 3.13 mm, and 7.53 ± 2.34 mm, respectively) (Fig. 6d). These data indicate that MSCs delivered in the CHS/ADM scaffold enhance vessel growth in wounds.

### Transplantation of CHS/ADM scaffold accelerates murine wound healing

**Fig. 7:**
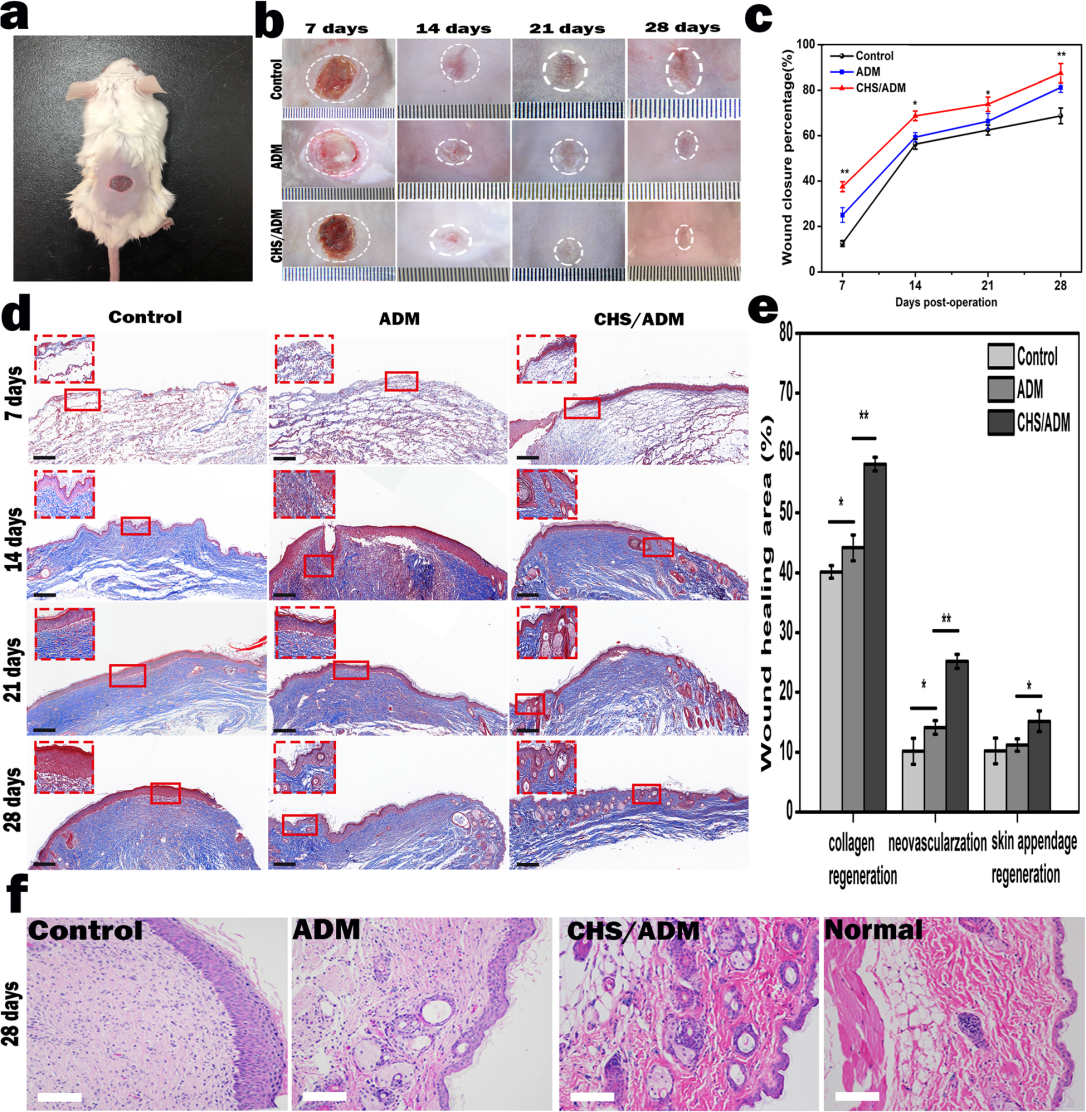
The CHS/ADM scaffold promotes skin reconstruction. **a** The full-thickness wound. **b** Wound closure and corresponding wound closure percentages after control, MSCs-ADM, or MSCs-CHS/ADM scaffold treatment on days 7, 14, 21, and 28. **c** Statistical analysis of the wound closure rates in continuous time (7, 14, 21, and 28 days). (**p < 0.01, *p < 0.05, *n* = 6) **d** Representative images of Masson’s trichome staining of the control, MSCs-ADM, and MSCs-CHS/ADM scaffold groups on 7, 14, 21, and 28 days postwounding (*n* = 6). Scale bar, 200 μm. Inset is × 400. **e** Quantitative analysis of collagen regeneration, neovascularization and skin appendage regeneration on days 14 (**p < 0.01, *p <  0.05, *n* = 6). **f** Hematoxylin eosin (H&E) staining of the healing wounds in the control group, MSCs-ADM scaffold group, MSCs-CHS/ADM scaffold group, and normal group (healthy mouse skin) on postoperative day 28. Scale bar, 100 μm

An 8-mm-diameter full-thickness murine excisional wound healing model was performed to evaluate the therapeutic efficacy of the MSCs seeded on CHS/ADM scaffolds in vivo (Fig. 7a). The ADM and CHS/ADM scaffold treatment groups showed obvious acceleration of wound closure at different levels compared with the control group (Fig. 7b, c). Notably, the CHS/ADM scaffold group exhibited better wound closure than the ADM scaffold group on the same postoperative day. Moreover, the wound area completely closed after 14 days, and almost no scar was found after 21 days in the CHS/ADM scaffold group (*p < 0.05). However, the wound was almost closed with a scab covering in the control group after 28 days. The Masson-stained sections showed that the transplanted MSCs in the CHS/ADM scaffold group had mature bundles of collagen deposition, while the control and ADM scaffold groups had disorganized collagen (Fig. 7d). Subsequently, collagen regeneration, neovascularization, and skin appendage regeneration were analyzed after 14 days (Fig. 7e), and the wounds treated with CHS/ADM scaffolds showed obviously more favorable than other groups. Moreover, hematoxylin eosin (H&E) staining indicated that the CHS/ADM scaffold group after 28 days appeared to be more similar to that of normal skin tissue than the control and ADM scaffold groups (Fig. 7f). Overall, these data demonstrate that MSCs delivered by CHS/ADM scaffolds markedly accelerated wound closure, reduced scar formation, and promoted wound repair.

## Conclusion

In summary, we developed a novel chitosan/acellular dermal matrix scaffold, which has favorable biodegradability and desirable biocompatibility, and particularly acts as an excellent ROS scavenger to address the challenge of transplanted cell survival by eliminating ROS during the inflammatory response. This scaffold provides a protective environment for cell survival in vivo and contributes to the vessel growth and the acceleration of wound healing, which demonstrates a great potential use in wound healing and other tissue regeneration applications.

## Supplementary information

**Additional file 1: Figure S1.** The effect of chitosan on MSCs differentiation without (*n*=6) and with H_2_O_2_ (30 μM) (*n*=5). Oil Red O staining of adipocytes and Alizarin red staining of osteoblasts at day 14 and 21, respectively. Scar bar: 20μm; **p < 0.01, *p < 0.05. **Figure S2.** SEM images showing the morphologies of the ADM and CHS/ADM scaffolds after 24 h degradation by collagenase. Scar bar: 10μm (n=5). **Figure S3.** Young’s modulus of the ADM and CHS/ADM scaffolds (n=6). **Figure S4.** The percent degradation mass of the ADM and CHS/ADM scaffolds after enzymatic degradation at different time points under ROS environment (30 μM H_2_O_2_) (n = 5).

## Data Availability

Not applicable.

## Abbreviations

ROS: Reactive oxygen species
CHS: Chitosan
CHS/ADM: Chitosan/acellular dermal matrix
MSCs: Mesenchymal stem cells
ADM: Acellular dermal matrix
ECM: Extracellular matrix
SEM: Scanning electron microscopy
PMNs: Polymorphonuclear neutrophils
DHE: Dihydroethidium
Calcein-AM/PI: Calcein-AM/propidium iodide
DAPI: 4′,6-Diamidino-2-phenylindole
PBS: Phosphate-buffered saline
RT-PCR: Real-time polymerase chain reaction
TPEF: Two-photon excitation fluorescence
SHG: Second harmonic generation
TPLSCM: Two photon laser scanning confocal microscope
GFP: Green fluorescence protein
SDS-PAGE: Polyacrylamide gel electrophoresis
LI-COR: Infrared Imaging System
α-SMA: Ascular smooth muscle actin
H&E: Hematoxylin and eosin
