# Supplementary material for: A barrier against reactive oxygen species: chitosan/acellular dermal matrix scaffold enhances stem cell retention and improves cutaneous wound healing
Source: Stem Cell Res Ther. 2020 Sep 7;11:383. doi: 10.1186/s13287-020-01901-6 (PMC7487689; doi:10.1186/s13287-020-01901-6)
Supplement: Supplementary file 1 — Additional file 1: Figure S1. The effect of chitosan on MSCs differentiation without (n=6) and with H2O2 (30 μM) (n=5). Oil Red O staining of adipocytes and Alizarin red staining of osteoblasts at day 14 and 21, respectively. Scar bar: 20μm; **p < 0.01, *p < 0.05. Figure S2. SEM images showing the morphologies of the ADM and CHS/ADM scaffolds after 24 h degradation by collagenase. Scar bar: 10μm (n=5). Figure S3. Young’s modulus of the ADM and CHS/ADM scaffolds (n=6). Figure S4. The percent degradation mass of the ADM and CHS/ADM scaffolds after enzymatic degradation at different time points under ROS environment (30 μM H2O2) (n = 5). [file 13287_2020_1901_MOESM1_ESM.docx]

**A Barrier Against Reactive Oxygen Species: Chitosan/Acellular Dermal Matrix Scaffold Enhances Stem Cell Retention and Improves Cutaneous Wound Healing** Wei Lin^a1,a2^, Xiaoyang Qi^b^, Wenjing Guo^c^, Danyang Liang^a1,a2^, Heting Chen^a1,a2^, Baoping Lin^a1,a2^, Xiaoyuan Deng^a,1a2^*

*a1 MOE Key Laboratory of Laser Life Science, College of Biophotonics & Institute of Laser Life Science, South China Normal University, Guangzhou, 510631, China*

*a2 Guangdong Provincial Key Laboratory of Laser Life Science, College of Biophotonics, South China Normal University, Guangzhou 510631, China*

*b The Brain Cognition and Brain Disease Institute of Shenzhen Institutes of Advanced Technology, Chinese Academy of Sciences, Shenzhen, 518055, China*

*c Guangzhou Institutes of Biomedicine and Health, Chinese Academy of Sciences, Guangzhou, 510530, China*

** To whom correspondence should be addressed. E-mail: xiaoyuandeng@scnu.edu.cn*

**Results and discussion**


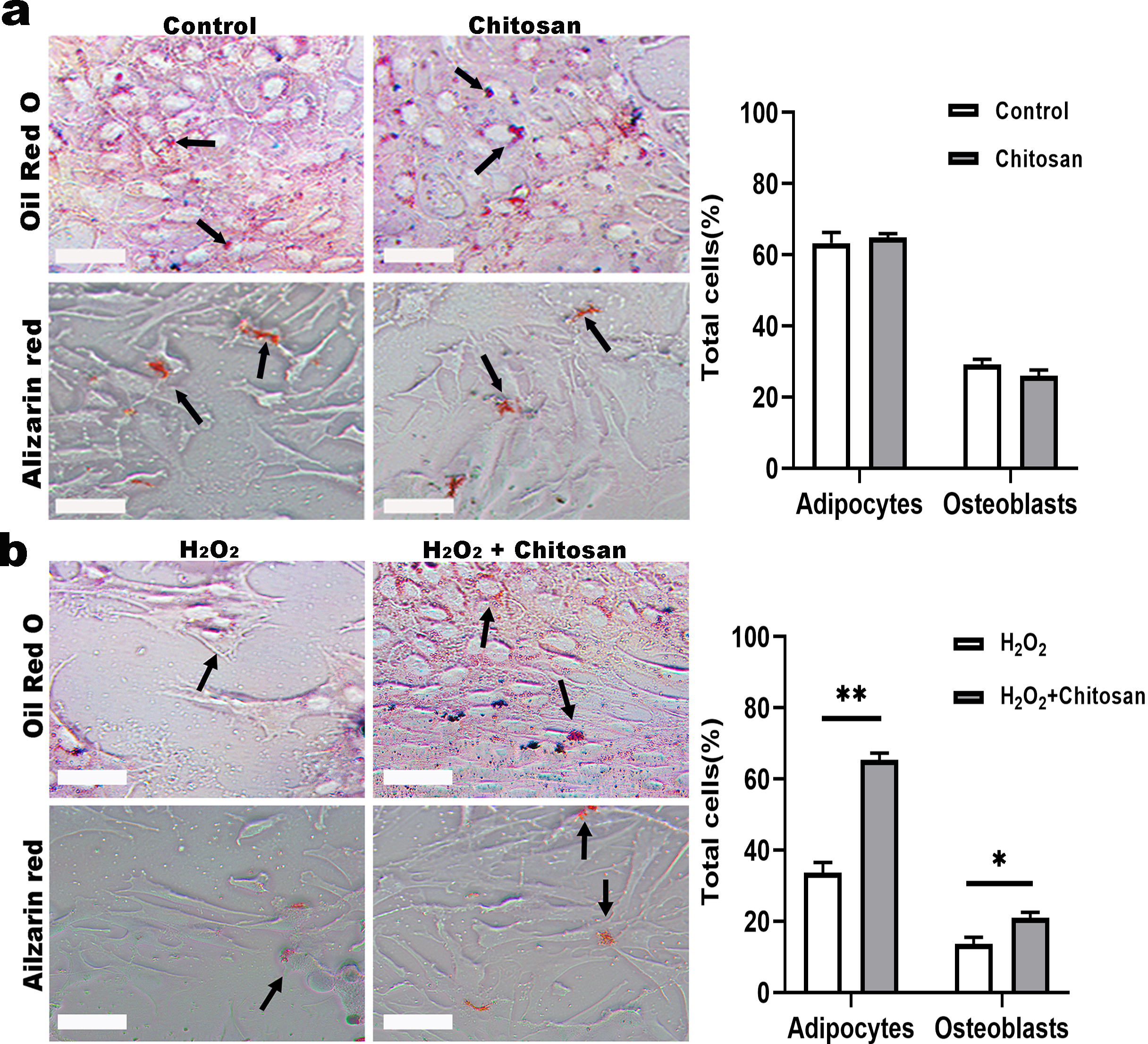


Figure S1. The effect of chitosan on MSCs differentiation without (n=6) and with H_2_O_2_ (30 μM) (n=5). Oil Red O staining of adipocytes and Alizarin red staining of osteoblasts at day 14 and 21, respectively. Scar bar: 20μm; **p < 0.01, *p < 0.05.


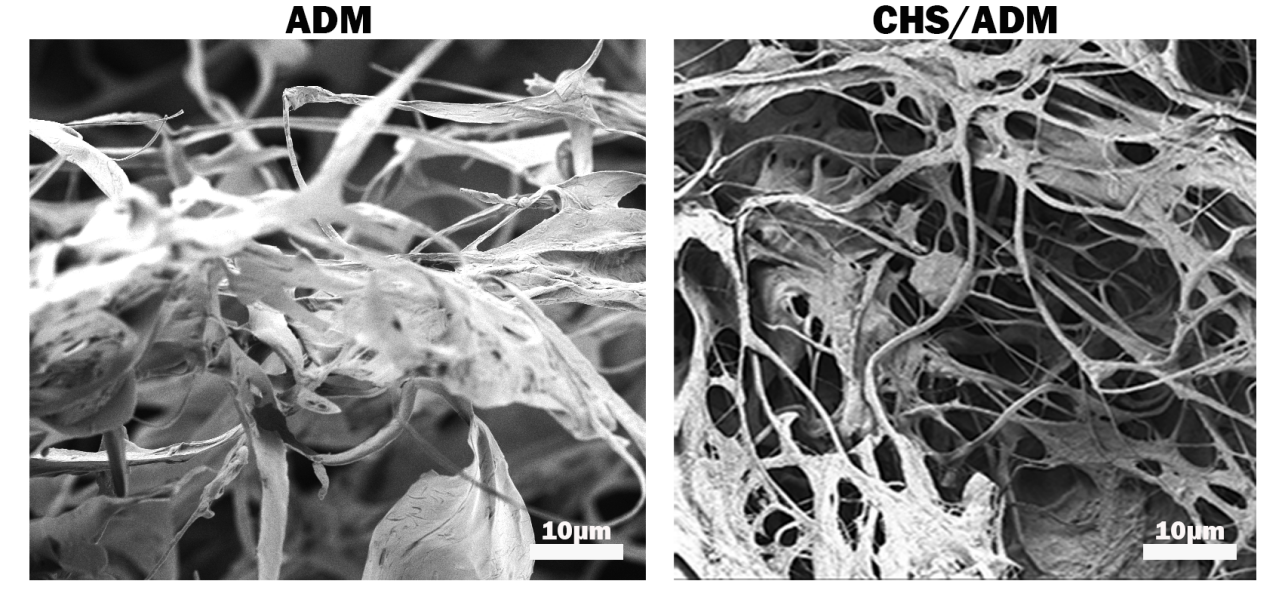


Figure S2. SEM images showing the morphologies of the ADM and CHS/ADM scaffolds after 24h degradation by collagenase. Scar bar: 10μm (n=5).


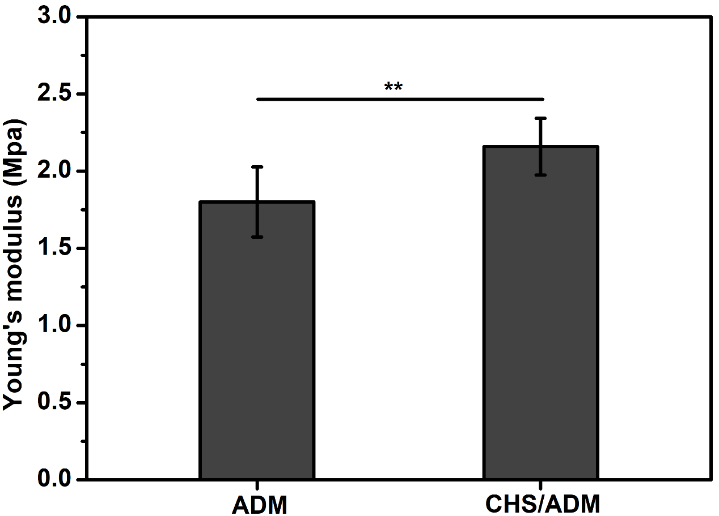


Figure S3. Young’s modulus of the ADM and CHS/ADM scaffolds (n=6).


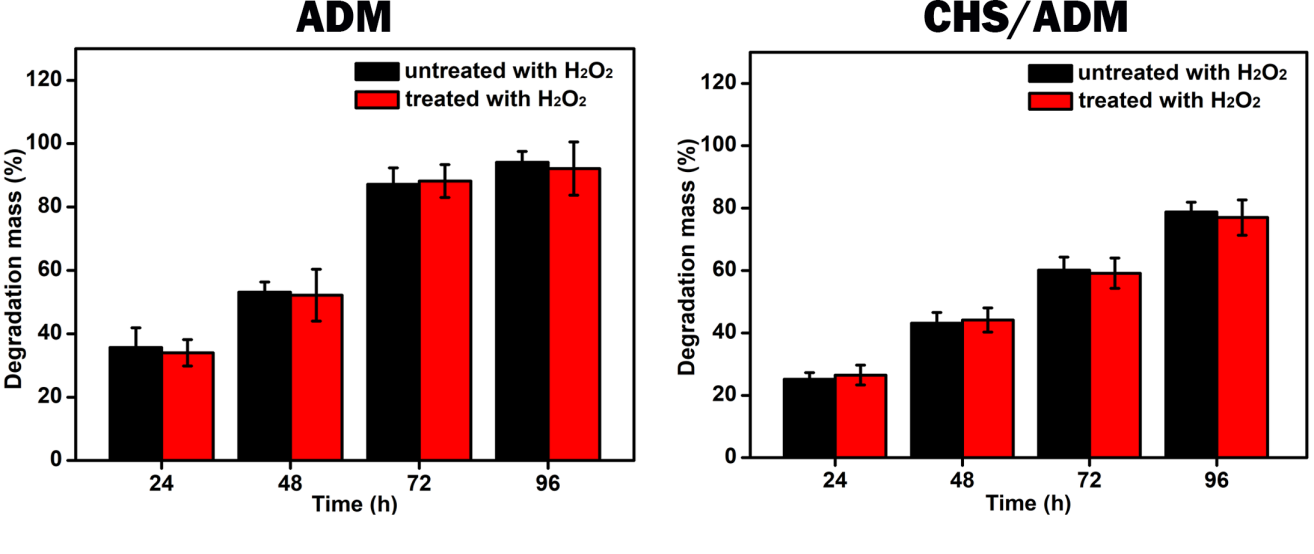


Figure S4. The percent degradation mass of the ADM and CHS/ADM scaffolds after enzymatic degradation at different time points under ROS environment (30 μM H_2_O_2_) (n = 5).
